# Primary pituitary tuberculoma with a pituitary apoplexy-like presentation

**DOI:** 10.1530/EDM-23-0092

**Published:** 2024-01-29

**Authors:** Gaayathri Krishnan, Nur Hidayah Mohd Makhatar, Tee Hwee Ching, Serena Khoo

**Affiliations:** 1 Endocrinology Unit, Department of Internal Medicine, Queen Elizabeth Hospital, Kota Kinabalu, Sabah, Malaysia

**Keywords:** Adolescent/young adult, Female, Asian - other, Malaysia, Pituitary, Pituitary, Infectious diseases, Pituitary apoplexy, Infectious diseases, Unique/unexpected symptoms or presentations of a disease, January, 2024

## Abstract

**Summary:**

Pituitary tuberculoma is extremely rare and may pose as a diagnostic challenge especially when encountered as an isolated lesion without other systemic manifestation of tuberculosis. A 21-year-old female was admitted for diabetic ketoacidosis. On the third day of admission following the resolution of diabetic ketoacidosis she developed a sudden onset of headache and blurring of vision suggestive of pituitary apoplexy. An urgent MRI brain revealed a large sellar mass with erosion into the sphenoid sinus and intracranial vasculitis. Transphenoidal surgery was done for tumour debulking which allowed histopathological examination of the sellar mass. Immunohistochemical examination of the sellar mass was positive for Gene Xpert MTB/Rif suggesting a tuberculoma. Anti-tuberculous therapy was commenced with full recovery of pituitary hormonal profile seen 7 months post-treatment. In regions with a high incidence of tuberculosis, a tuberculoma should be a considered in a diagnostic evaluation of a sellar lesion.

**Learning points:**

- In an endemic area of tuberculosis, tuberculoma should be considered as a differential diagnosis when evaluating sellar lesions.
- Pituitary tuberculoma can present with pituitary apoplexy-like symptoms.
- Prompt diagnosis and treatment may lead to recovery of pituitary function.

## Background

Pituitary adenomas remain the most common cause of lesions affecting the sellar region. Cysts, malignancies and infections account for less than 10% of sellar lesions but are important differentials that need to be considered when evaluating these lesions (1). Among these, primary pituitary tuberculoma is extremely rare with fewer than 110 cases reported in the literature thus far (2). Since the development of anti-microbial therapy, tuberculomas have accounted for 0.15–4% of intracranial lesions, mainly in developing countries (3). Primary pituitary tuberculoma has often been mistaken for an adenoma owing to its non-specific clinical and radiological findings. Rarely, these pituitary tuberculomas can present with symptoms of pituitary apoplexy. Pituitary apoplexy is an emergency condition characterized by acute onset of headache, loss of vision, altered mental status and hormonal deficiencies in particular secondary adrenal insufficiency, which can be life-threatening (4). Here, we report a case of a young female who presented with symptoms of pituitary apoplexy and was found to have partial hypopituitarism secondary to an isolated pituitary tuberculoma. She was treated with anti-tuberculous therapy and showed good clinical and biochemical recovery.

## Case presentation

A 21-year-old female was initially admitted with a history of vomiting and lethargy for 3 days and was treated for diabetic ketoacidosis. She was previously diagnosed with type 2 diabetes and was treated with oral anti-diabetic medication and basal insulin. She had missed her medications 3 days before admission. She was commenced on intravenous insulin and saline as per local guidelines for management of diabetic ketoacidosis. Her symptoms along with her biochemical parameters improved and diabetic ketoacidosis resolved within 24 h of admission. She was monitored inpatient for optimization of her blood glucose levels, however, during her third day of admission, she complained of sudden loss of vision in both eyes and severe headache. She also had a new onset of fever with a documented temperature of 37.8°C. Prior to this, the patient was afebrile in the ward. At the onset of headache and vision loss, there was no documentation of hypotension or ketonemia. She did not complain of any fever or headaches prior to admission. There was no other neurological deficit aside from the vision loss. There was also no history of trauma preceding the symptoms, and she had no history of cough or tuberculosis (TB) contact. On further history, she complained of oligomenorrhoea for the last 2 years. There was no history to suggest hypofunction or hyperfunction of other pituitary hormones. On examination, she was alert and conscious with a full Glassgow coma scale. Bitemporal hemianopia was noted on visual field assessment, and no papilledema was present on fundoscopy. The visual acuity of her right eye was 6/7.5 and her left eye was 6/7. Other cranial nerves were intact. Upon peripheral neurological assessment, power was full over all limbs, tone and reflexes were normal. There was no neck stiffness; Kernig’s and Brudzinski’s signs were negative. There were no features to suggest Cushing’s syndrome or acromegaly.

## Investigation

**Figure 1:**
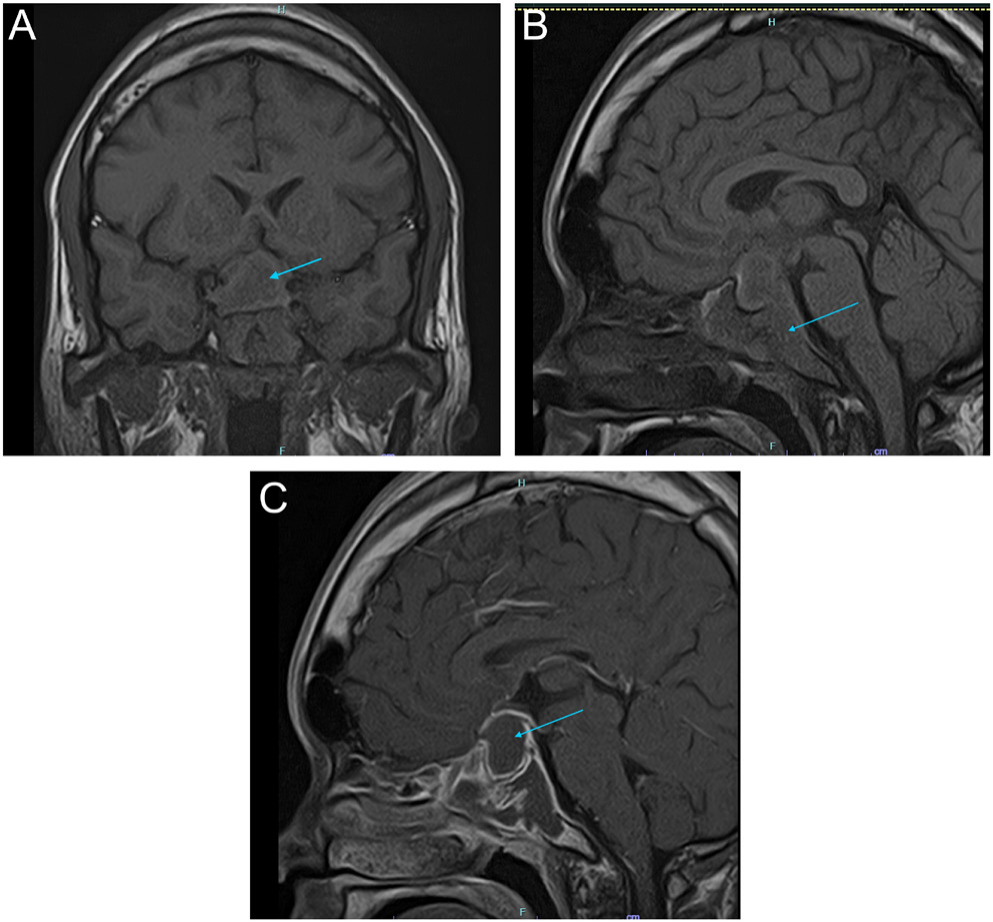
(A) T1-weighted coronal image pre-contrast – isointense sellar mass with erosion into bilateral cavernous sinus. (B) T1-weighted sagittal image pre-contrast – isointense sellar mass with erosion into sphenoid sinus and clivus. (C) T1-weighted sagittal image – peripheral enhancement seen post-contrast.

The patient’s routine hematological and biochemical investigations were normal (Table 1), HIV and blood cultures were negative, and her chest X-ray was clear. Otolarnygoscopy was performed and yellow discharge at the posterior aspect of the nasopharynx was found, raising the suspicion of an infective aetiology; however no cultures were sent. MRI brain with pituitary protocol was done which revealed a sellar lesion measuring 1.5 cm × 2.6 cm × 2.0 cm, with erosion into the sellar turcica and medial wall of the sphenoid sinuses, superiorly impinging the optic chiasm. There was no bleed seen within the lesion. The lesion demonstrated iso- to hyperintense signal on T1W and iso- to hypo-intense signal on T2W. Peripheral enhancement was seen post contrast (Fig. 1). Additionally, magnetic resonance arteriography (MRA) brain was done, which showed beaded appearance of the supraclinoid region of both internal carotid arteries, proximal middle carotid artery (M1) as well as A1 segment of right anterior cerebral artery suggestive of vasculitis. Hormonal assays done preoperatively revealed hypogonadotropic hypogonadism and secondary hypothyroidism (summarized in Table 2). She was empirically started on hydrocortisone prior to the commencement of thyroxine. She then underwent endoscopic transsphenoidal hypophysectomy and tumour debulking. The procedure was uneventful with no surgical related complications. Post operatively, she developed transient arginine vasopressin deficiency (AVP-D) which was treated with desmopressin. Cerebrospinal fluid (CSF) examination analysis was not sent. Histopathological examination of the sellar lesion revealed only necrotic tissue, with no presence of granuloma or caseating necrosis. There was no pituitary tissue seen and staining for acid fast bacilli (AFB) was negative. Fungal and bacterial cultures were negative. Immunohistochemistry testing of the tissue using Gene Xpert MTB/Rif was positive with indeterminate rifampicin resistance, which suggested a tuberculoma.

**Table 1:** Key haematological and biochemical investigations.

Investigations	Results	Reference range
White blood cells, 10^9^/L	10.9	4–10
Haemoglobin, g/dL	12.1	12.0–15.0
Haematocrit, L/L	37.2	36–46
Platelet count, 10^9^/L	407	150–410
C-reactive protein, mg/L	1.0	<5
Sodium, mmol/L	137	135–145
Potassium, mmol/L	3.9	3.4–4.5
Urea, mmol/L	2.6	2.8–8.1
Creatinine, µmol/L	66	59–104
Total bilirubin, µmol/L	14	<22
Alanine transaminase, U/L	15	<34
Aspartate transaminase, U/L	16	<45
Alkaline phosphatase, U/L	160	35–105
Total protein, g/L	88	64–83
Albumin, g/L	47	35–52

**Table 2:** Summary of hormonal investigations.

Hormone assays	Reference range				On admission	Post ATT at	
	Normal range	FP	MC	LP		3 months	7 months
LH, mIU/mL		1.80–11.78	7.59–89.08	0.56–14	0.3	3.5	2.91
FSH, mIU/mL		3.03–8.08	2.55–16.69	1.38–5.47	1.2	5.4	5.1
Prolactin							-
µg/L	5.2–26.5				1.1	9.1	
ng/mL	5.2–26.5				1.1	9.1	
Testosterone							-
nmol/L	0.48–1.85				1.2		
µIU/mL	13.8–53.3				34.16		
Estradiol, pmol/L		77–921	139–2382	77–1145	<37	< 37	109
FT4							
pmol/L	12–22				8.95	14.19	18
ng/dL	0.93–1.55				0.69	1.09	1.39
TSH							
mIU/L	0.4–4.0				2.03	1.6	1.8
µIU/mL	0.4–4.0				2.03	1.6	1.8
Cortisol							
nmol/L	101.2–535.7				175.7		
µg/dL	3.67–19.41				6.32		
SST*							
0 min							
nmol/L						255	334
µg/dL						9.18	12.02
30 min							
nmol/L						409	482
µg/dL						14.72	17.35
60 min							
nmol/L						440	515
µg/dL						15.84	18.54
Treatment					Started HC, T4, PG	T4 and HC reduced, PG stopped	T4 and HC stopped

*Above 450 nmol/L is considered adequate response.

ATT, anti-tuberculosis therapy; FP, follicular phase; FSH, follicle stimulating hormone; FT4, free thyroxine; HC, hydrocortisone; LH, luteinizing hormone; LP, luteal phase; MC, mid-cycle; PG, progyluton; SST, short synacthen test; T4, thyroxine; TSH, thyroid-stimulating hormone.

## Treatment

She was started on anti-tuberculous therapy with isoniazid 5 mg/kg body weight, rifampicin 10 mg/kg body weight, pyrazinamide 25 mg/kg body weight and streptomycin 15 mg/kg body weight along with pyridoxine supplementation (20 mg/day) for 2 months followed by isoniazid and rifampicin for 10 months. She also received tapering doses of dexamethasone for a total of 6 weeks for the treatment of cerebrovascular tuberculosis.

She was discharged 1 week post-surgery with tablet thyroxine 75 µg daily, tablet hydrocortisone 15 mg daily in divided doses and oral cyclical estradiol 2mg and norgestrel 0.5 mg.

## Outcome and follow-up

**Figure 2:**
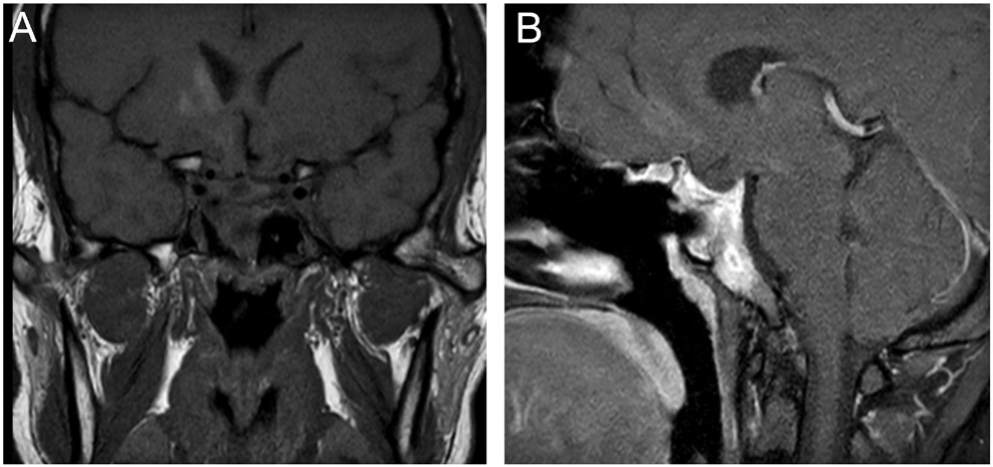
(A) T1-weighted coronal image pre-contrast – previously seen rim-enhancing lesion is not seen in this study. (B) T1-weighted sagittal image shows residual peripherally enhancing lesion at the body of clivus.

Repeated MRI pituitary done after 2 months of intensive therapy revealed a residual enhancing lesion measuring 0.3 × 1.1 × 0.8 cm and previously seen sellar lesion was no longer visualised (Fig. 2). Beaded appearance of bilateral internal carotid arteries was no longer visualised, while the proximal middle carotid artery (M1) as well as A1 segment of right anterior cerebral artery remained similar suggesting partial resolution of the vasculitis.

**Figure 3:**
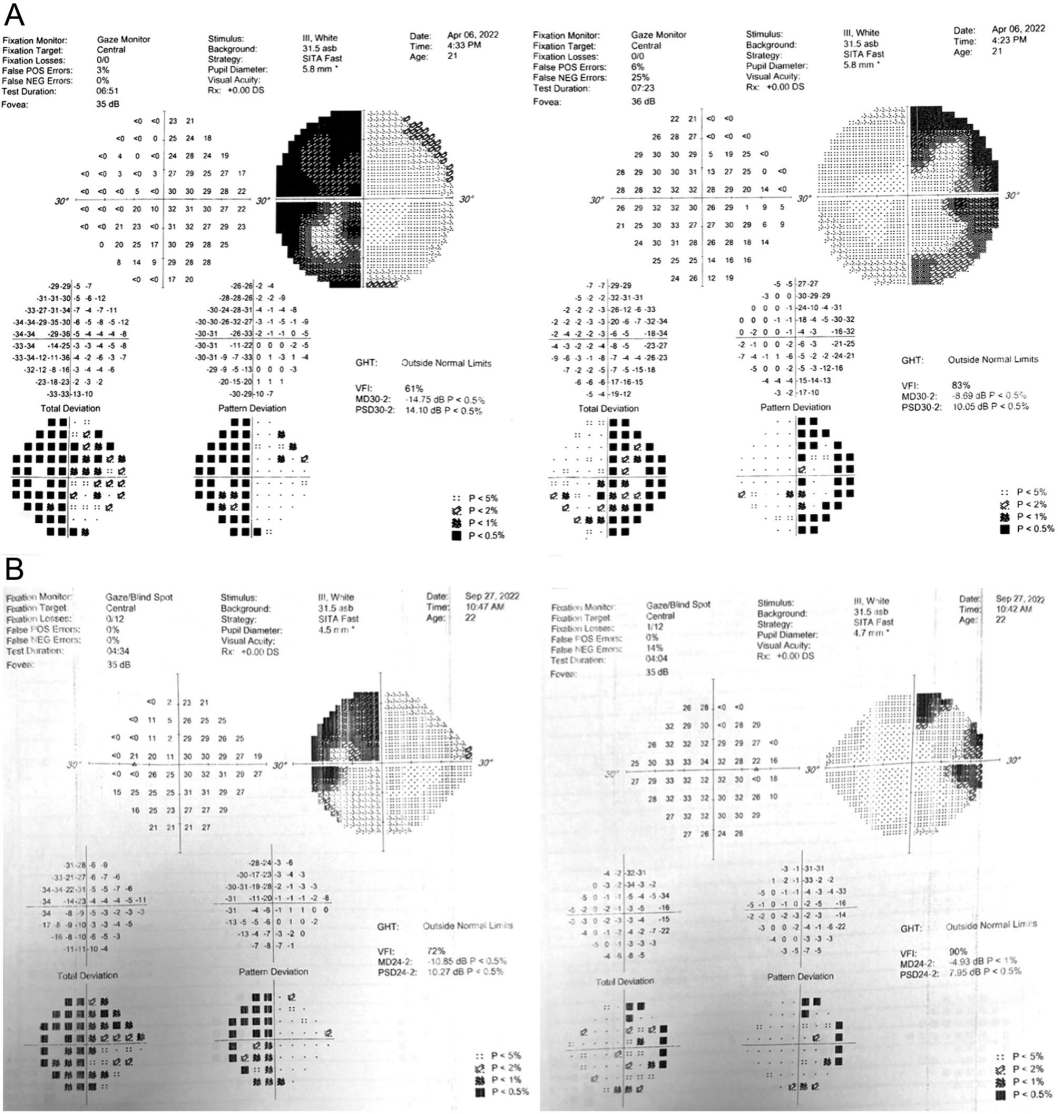
(A) Humphreys visual field test of the bilateral eyes done pre-operatively. (B) Humphrey’s visual field test of the bilateral eyes done 5 months post-operatively.

At 7 months of anti-tuberculous therapy, her hormonal profile showed full recovery and she was able to discontinue her thyroxine, cyclical estradiol and norgestrel and hydrocortisone supplementation. There was also marked improvement in her bitemporal hemianopia seen on Humphrey’s test done 5 months post-operatively (Fig. 3). She had also regained a normal menstrual cycle.

## Discussion

Tuberculosis remains a major cause of mortality and morbidity worldwide, with a total of 9.9 million people affected in the year 2020. South East Asia contributes to 43% of the total disease burden which is the largest globally (5). A recent meta-analysis showed a prevalence of 9% of CNS TB among hospitalized patients, with high mortality rates of approximately 42% (6). Tuberculosis occurs through inhalation of the bacilli which disseminates via a hematogenous spread. CNS TB originates from the formations of tuberculous foci also known as Rich foci in different locations which eventually present as a tuberculoma, meningitis or tuberculous abscess (7). Direct invasion through the paranasal sinus is another possible mode of entry; involvement of the sphenoid sinus was seen with our patient and 6 of 18 patients with pituitary tuberculosis reported by Sharma *et al.* (3). A presentation of pituitary apoplexy is seen in approximately 8–10% of patients with pituitary adenoma, with tuberculoma being an extremely unusual cause. The reason for such a presentation of a tuberculoma is possibly due to infarction or bleeding within the lesion secondary to inflammatory vasculitis (4). In such cases, surgery is usually indicated for urgent decompression, and this allows for histopathological evaluation to ascertain the diagnosis. In a case report by Verma R *et al.*, the patient with apoplexy-like presentation was managed conservatively as diagnosis was ascertained by CSF analysis obtained via a lumbar puncture, that was positive for TB PCR (4). If surgery is opted for, transsphenoidal approach is preferred to reduce CSF contamination (3, 4). Symptoms related to endocrinopathies that were commonly seen in patients with a sellar tuberculoma were galactorrhoea, amenorrhoea and polyuria secondary toAVP-D. As observed in cases of other infections, this is likely due to the systemic spread of tuberculosis through the bloodstream leading to involvement of the posterior pituitary and the pituitary stalk rather than the anterior pituitary. In the largest case series of 18 patients with pituitary TB, there was a female preponderance of (13/18) with a galactorrhoea–amenorrhoea complex seen in 4 of 13 patients (3). While others have more commonly reported hyperprolactinemia likely due to stalk disruption, in our patient, low levels of prolactin levels were seen suggesting lactotroph dysfunction (1, 3). Radiologically, MRI is the mainstay of imaging to diagnose pituitary lesions; however, it is difficult to differentiate pituitary TB from other lesions due to its non-specific imaging findings. They are mostly seen as isointense to hypointense on T1-weighted images and hyperintense in T2-weighted images with enhancement post-contrast. Some cases have demonstrated ring enhancement, stalk thickening and dural enhancement (4, 8). Additionally, MR spectrometry can be helpful, as a lipid peak at 1.13 ppm is suggestive of tuberculoma (8). In our case, there was presence of vasculitis in addition to the sellar mass. Tuberculous vasculitis usually occurs as a complication of TB meningitis and accounts for up to 12.4% of secondary vasculitis. The arteries that are most commonly affected by tuberculous vasculitis are those located at the base of the skull, notably the middle cerebral artery (9). Histopathological examination with demonstration of the mycobacterium confirms the diagnosis and the presence of caseating granuloma is characteristic of tuberculosis. Given the paucibacillary nature of CNS TB, in most cases, AFB was not seen on direct smear (5). In our case, the diagnosis was made based on positivity of Gene Xpert MTB/Rif. The pooled sensitivity and specificity of gene expert in tissue samples are 81.2% and 98.1% respectively and may be used as an additional testing in patients with suspected pituitary tuberculoma (10). In a literature review of pituitary tuberculoma, 21 of 32 patients showed improvement of their endocrinopathies with anti-tuberculous therapy (8). In another case series, most patients showed improvement in their pituitary function post anti-tuberculous therapy for a total of 18 months (9). In conclusion, pituitary tuberculoma is very rare presentation of CNS tuberculosis and requires a high clinical suspicion; presence of cerebral vasculitis and involvement of sphenoid sinus may aid in differentiating a tuberculoma from an adenoma. This disease shows a good clinical response with anti-tuberculous therapy especially when initiated early (9).

## Declaration of interest

The authors declare that there is no conflict of interest that could be perceived as prejudicing the impartiality of the case study reported.

## Funding

This work did not receive any specific grant from any funding agency in the public, commercial or not-for-profit sector.

## Patient consent

Written informed consent for publication of their clinical details and clinical images was obtained from the patient.

## Author contribution statement

GK and NHMM were the named physicians for the patient and wrote the case report and performed literature reviews for the discussion. THC is the endocrinologist involved in management of patient and provided suggestions for further improvement. SKSK is the consultant endocrinologist of this patient and provided suggestions for further improvement.
